# Online Indicators of Written Production: ‘Bio-Behavioural’ Markers of Dyslexia–Dysorthographia?

**DOI:** 10.3390/brainsci14111125

**Published:** 2024-11-07

**Authors:** Audrey Mazur, Matthieu Quignard

**Affiliations:** 1 Laboratoire d’Excellence Advanced Studies on Language Complexity ASLAN, Université de Lyon, 69342 Lyon, France; 2 Laboratoire CNRS ICAR, UMR5191, CNRS, Université Lyon 2 et ENS de Lyon, 69342 Lyon, France; matthieu.quignard@ens-lyon.fr

**Keywords:** dyslexia, higher education, handwritten production, online indicators

## Abstract

Dyslexia–dysorthographia is a neurodevelopmental disorder in which the symptoms appear during the person’s development (generally around the age of 7 or 8) and persist throughout life. The study of this written language disorder mainly focuses on children, principally in the clinical, cognitive science and neuroscience fields. The importance and originality of this study are that it investigates the impact of dyslexia–dysorthographia on written production in young adults (students) with dyslexia, from a psycholinguistic perspective. To do this, students and matched-control individuals were asked to produce written texts on the same theme. These productions were then analysed, observing on-line indicators, such as pause location and duration. The current investigation found that students with dyslexia still have important difficulties with writing and their lack of automation in spelling has consequences on the transcription and planning process: although they have the same handwriting speed, pressure and word rate as control students, they make longer pauses before words, especially before short and long words, words involving grammatical inflexion, grammatical words and punctuation.

## 1. Dyslexia, Writing and Biomarkers

Dyslexia, which is associated with dysorthographia, is a written language disorder involving difficulties in reading and writing. Its diagnosis is based on behavioural indicators assessed using standardised test batteries. In parallel with this, the technics used in the domain of brain imaging are growing more and more, which enables us to have a better understanding of structural brain differences that exist between people with and without dyslexia [1]; we are speaking about biomarkers. There exist several methods for studying biomarkers of dyslexia, among them, “standardized psycho-educational tests, web-based/mobile-based games, eye-movement tracking, MRI and EEG scans, MEG scans, PET scans, video and images captured during cognitive/phonological tasks” [2] (p. 10). Biomarkers are characteristics objectively measured “as indicators of normal biological processes, pathological changes, or pharmaceutical responses to a therapeutic intervention” [2,3]. The European Society of Radiology [3] references three types of biomarkers—biochemical or histological parameters; biochemical parameters or cells; and anatomical, functional or molecular parameters. Biomarkers have multiple applications, such as 1—prediction of disease risk; 2—detection: identification of patients with disease; 3—staging: classification of the extent of disease; 4—grading: use as an indicator of disease aggressiveness and prognosis; and 5—assessment of response to treatment [3] (p. 42).

Written production provides biomarkers that enable us to observe this complex activity. These biomarkers are totally linked to the writer’s tools, in that they are all actions linked to pencil movements: pressure, pencil lift, duration, length of the pencil traces, etc. These elementary biomarkers enable researchers to focus more specifically on the writers themselves and avoid the biases of cognitive interpretations. Taking these biomarkers as a basis, indicators at a more abstract level, which enable the observation and analysis of the individual’s written production, are set up. These indicators are linked to a model of the motor and cognitive aspects of written production (for this study, see Haye and Flower, among others, see Section 3.1). Among them, we can find on-line indicators, such as pause duration, pause location, fluency, word rate or speed during a written task. 

The observation of these on-line indicators allows us to have a better idea of the impact of dyslexia on written activities. Indeed, previous studies have shown, for instance, that students with dyslexia have a handwriting dynamic that is significantly different from a matched-control population [4]: for example, they make longer pauses before punctuation marks or inside a word. The written bio-markers to which these indicators are linked can therefore predict and help detect dyslexia, as well as help assess response to treatment. Like eye-movement tracking, these actions, which are directly linked to pencil movements in written productions, can be used to help classify learners into individuals with or without dyslexia.

This study sets out to examine biomarkers of the handwriting dynamics of students with dyslexia compared with matched-control students, in order to better understand the impact of dyslexia on written productions and to detect what on-line indicators are sufficiently strong to predict, and help to detect and assess response to treatment. Students with and without dyslexia were asked to produce written narrative and expository texts, in an ecological context, about problems between people. The study offers some important insights into dyslexia in adulthood and fills a gap in the research on this matter. Indeed, there is still very little work on adults with dyslexia [5,6,7,8,9,10], and this is especially true of the manifestations of dyslexia in spontaneous language production (particularly in writing); current work on adults with dyslexia is mainly in three areas: clinical, cognition and neuroscience [7].

## 2. Definition of Dyslexia and Persistent Difficulties in Adulthood

According to the DSM-5 [11], dyslexia is defined as a specific learning disability, classified as a neurodevelopmental disorder. Specific learning disorders group together all the signs of reading, writing and arithmetic disorders under common diagnostic criteria. They are defined as skills that are significantly below those expected for a given chronological age, with a significant negative impact on academic performance at school or university. The disorders are manifested by difficulties in learning and using academic skills for at least 6 months, and are reflected in at least one of the following symptoms: inaccurate, slow or laborious word reading; difficulty in understanding the meaning of what is read; difficulty in spelling; difficulty in written expression; difficulty in mastering number sense, numerical facts or arithmetic; and difficulty in mathematical reasoning. These developmental hindrances, which appear during the years of schooling, can only manifest themselves when the demands exceed the limited capacities of the individual, and they cannot be explained by other impairments (intellectual, hearing, visual, etc.) [10,12]. Dyslexia appears to be the most frequent manifestation of specific learning difficulties, and refers to “a learning profile characterized by difficulties in recognizing common words accurately or fluently and poor decoding and spelling skills” [10] (p. 2, translated from the DSM-5).

Dyslexia, as a neurodevelopmental disorder, endures throughout life, and adults with dyslexia still display persistent difficulties in reading and writing [among others, [13,14,15,16,17,18,19,20,21]. International studies show that adults with dyslexia can have lexical difficulties, such as confusion between monosyllabic words (like *which* and *with*), omission of words in sentences, use of unexpected vocabulary, or production of shorter words, when compared to control adults [13,18,19,21]. Recent French studies qualify these results by revealing that students with dyslexia produce the same types of word as control students, regardless of the spelling consistency and the number of letters or syllables in the words used [22], but make more spelling errors, which is in line with the findings of previous international studies in English [13,16,19], Spanish [15] or French [23,24,25,26]. Moreover, international literature also concludes that adults with dyslexia have persistent difficulties with syntax [13,23,24] and punctuation [26], and they do not revise their own texts as efficiently as control students [27,28,29].

Although it is difficult to give a stable prevalence of dyslexia in France and worldwide, the fact remains that this specific learning disability affects a large proportion of our fellow citizens. Indeed, prevalence for dyslexia can vary from one study to another, so average numbers are used. According to Inserm [30], 7% of children in France have this type of disorder; another report states that between 5 and 10% of children aged between 8 and 12 are dyslexic [31]. According to the DSM-5 [11], the figures are much higher: between 12 and 15% of the school-age population. For the adult population, it is even more complicated to say, as statistics are even rarer [10] and we have no large-scale epidemiological studies [6]. The DSM-5 refers to 4% of the adult population and in France it is estimated that this proportion may be between 6 and 8% [6,32,33]. Although prevalence cannot be clearly established, the fact remains that dyslexia persists with age and is in fact “a non-transitory developmental deficit, which makes it possible to envisage the reported prevalence rates in adulthood” [6] (p. 5). That said, since the 2000s and the Ringard report [34] and the subsequent action plan, more children are detected at an earlier stage and therefore diagnosed earlier. Whether as a direct consequence or not, the proportion of dyslexic students in higher education increases every year [35]: dyslexic people, therefore, achieve a degree of academic success, but it is not without difficulties. Indeed, early diagnostic, care and support is a strong protective factor [36], but this disorder persists throughout life and the resulting difficulties do not disappear [37], despite remediation. Although [36] talks about the strong benefits of treatment, she also concludes that one of the limitations of this treatment lies in the spelling difficulties complained of by most dyslexic adults.

## 3. Written Production and Dyslexia

### 3.1. Written Production 

Writing is a very costly and complex cognitive task involving various cognitive processes [37,38]: planning (generating ideas); translating (developing linguistic and graphic structures from internal representations); and reviewing (performing control operations on the text). The writing processing system is described as a capacity-limited system (see [26], for a more descriptive explanation): when one allocates more cognitive resources to transcription, fewer resources are available for higher-level processes such as seizing ideas and organising them [39]. Writing also entails the following [40,41]: 1—the treatment of several linguistic and conceptual dimensions during the graphic realization; 2—the cost of the transcription step, regardless of how small it is and; 3—the possible impact of the cognitive resources allocated to the low-level processes on high-level processes. As regards the spelling process, it becomes automated with age and experience, and it is only between 9 and 12 that children with typical development automate conversion from phonemes to graphemes and the motor processes linked to the graphic activity [42]. Indeed, after the age of 10, handwriting becomes an autonomous skill, independent of spelling abilities [43,44]. From this age onwards, the dynamics of writing is no longer associated with the graphomotor aspects of written production, and proprio-kinaesthetic skills are acquired [45]; these specific skills promote awareness of movement [46], including graphomotor movement. Indeed, a deficit in proprio-kinaesthetic skills affects the automation of the writing gesture, as children adopt an inefficient pencil grip and compensate by exerting greater pressure on the pen [45]. But it is not before the age of 16 that adolescents can totally manage all aspects of written production, including the planning process [47]. Previous studies reveal that the quality and length of written texts depend on the automation and management of spelling conversion [42]. Indeed, if spelling conversion becomes a low-level process, cognitive resources can be allocated to higher-level processes, such as a high-level planning strategy to organize their text. Moreover, it seems that the high cost of the spelling dimension, due to a lack of automation, results in “poor compositional performances” [48] (p. 397). 

The fact is that people with dyslexia do not totally automate spelling conversion [42] and this impacts their written productions: more spelling errors, difficulties in handling punctuation, etc. This lack of automation also has an impact on the on-line dynamic of the written activity, such as handwriting speed [49], which impacts the amount of text produced, but also its quality [50,51,52,53,54,55]. People with dyslexia who have not automated spelling are very disadvantaged [56,57]. During a writing activity, they have to carry out different operations (organizing their ideas, translating them into words in accordance with the rules of the writing system), and are faced with a number of constraints (types of text to produce, instructions, etc.). Non-automated spelling conversion entails the mobilization of cognitive resources to handle the process, and the direct consequence of this is that fewer cognitive resources are available for high-level processes. 

### 3.2. Methods to Analyse Written Production and the Writing Dynamic

To have a better understanding of written language production, researchers in psycholinguistics can employ two types of methodologies: (a) an off-line method and (b) an on-line method. Off-line analysis, which is the oldest method and has led to the development of most production models [58], focuses on the end products of language production, for instance by observing lexical and syntactic choices or errors. On-line analysis entails taking into consideration the processes involved in language activity in real time by studying behavioural indicators, such as pauses, flow or verbal protocols [58,59], which make an important contribution to the understanding of the mechanisms involved in language production [40]. As said previously, during written activities different on-line indicators can be observed, such as pause duration. The first studies on pauses in written production date from the late 1970s, with the works of Flower and Hayes [38,60] and Williams, [61], for example. Pauses were described as an indicator similar to eye fixation during reading [62]. It appears to be a natural trace, since the interruption of graphomotor activity occurs without experimental intervention, and it is an easily observable trace: a pause occurs every time the writer raises his/her pencil and thus stops writing. This early work required very precise observations by the experimenter, who had to record (chrono-video) the writer while he/she was writing and then watch the films and time all the pauses (see, for example, the work of Matsuhashi [63]). The advent of graphic tablets [64] has made it possible to make greater use of pauses, as well as other indicators of written production such as pressure, handwriting speed, etc.

Concerning the observation of pauses, two aspects are studied: their duration and location in the written text [65]. The duration of pauses is different according to their syntactic location. The longer the unit, the longer the pause [65,66,67,68], even if before certain unites like punctuation and connectors pauses do have a longer duration because they involve considerable syntactic planning [66]. For instance, pauses are longer at the boundaries of large syntactic or textual units (sentences, paragraphs) than at lexical boundaries [64]. In this sense, the length of pauses between paragraphs and sentences is mainly a result of knowledge management, and therefore conceptual planning, even if, at the beginning of sentences syntactic and lexical processes may be involved in conjunction with conceptual planning. Moreover, pauses between propositions would indicate linguistic formulation and pauses between words would be linked to lexical processes, such as vocabulary choices [64,69,70]. The analysis of pauses can be completed by the observation of bursts, which can be defined as execution periods, defined as the time elapsed between two pauses during which the writer has produced at least one word [64]. This indicator allows to observe the writing process, to describe and analyse cognitive operations (planning, transcription, etc.) [68]. This unit is defined as the average length of text written between two long pauses [68,71,72] and the idea is to see if writers have the skills to produce a long series of words without long pauses. The length of bursts may reflect writing skills: expert writers compose their texts with longer execution periods, translating fluidity in their production [68,71,72,73,74]. Moreover, when motor execution is automated, planning, texting or revising can be implemented while the writers transcribe their text, although linguistic formulation of the text is most frequently used [75,76,77,78,79]. Consequently, studying bursts or the syntactic structures that they contain can provide important information about writing strategies [64].

Concerning flow and the dynamic of writing, handwriting speed, pressure and word rate can be observed. Handwriting speed provides information about the writer’s writing dynamic. It corresponds to the total distance of tracings divided by the writing time in cm/s, without taking into account pauses or movements in the air [50,68,80]. During the writing activity, changes in the handwriting speed may reflect planning or difficulties in elaborating one’s text [41,81,82]. Pressure corresponds to the pressure that the writer exerts on the pen when he/she is writing, i.e., the pressure exerted by the pen on the surface of the tablet [45]. This indicator is directly linked to the graphomotor skills. Word rate, defined as the number of linguistic units (words, letters, syllables) produced in a given period of time (minutes or seconds), is rarely observed in studies on on-line indicators [40], but it can be impacted by the cost of the transcription as well as by a spelling conversion system that is not totally automated. For adult writers, an increase in the cognitive load on the conceptual and linguistic levels can result in a slowdown of the word rate [49], which is also sensitive to the degree of the accessibility of information in memory [70]. Another criterion is familiarity with the theme of the text: the more familiar the theme, the faster the writing speed [49,82].

A link between the nature of the processes and the variation in the indicators is therefore possible [65,69,70]. So, for example, the longer a pause, the more costly the cognitive activity correlated with it. Depending on its duration, and also its place, we can then deduce that the speaker/writer plans an entire paragraph by looking for relevant information, diagrams and plans [65,69,70]. Moreover, this type of analysis helps to explain the written processes and the difficulties of typical and atypical writers [45,68,80]. Finally, it seems obvious to link the two observation methodologies in textual psycholinguistics: on-line and off-line, which are essential keys to a full understanding of the production phenomenon and to understanding where the difficulties lie. Indeed, “analyses of pause locations and durations, combined with textual description, serve as a window to the cognitive framework underlying text production processes” [83] (p. 21). To finish, an important fact should be stressed for the present study: handwriting speed is no longer associated with the graphomotor aspects of written production [43,44]. We can also observe on-line indicators without a possible impact of graphomotor gestures. 

In this present study, we propose to examine some on-line indicators, such as pressure, handwriting speed, word rate and the duration and location of pauses, also taking into account some off-line indicators like grammatical class, length, frequency and the phoneme/grapheme consistency of words associated with the observed pauses (see Part 5.4 for a definition). Studies focusing on on-line indicators are rare, even more so those combining observations of on-line and off-line indicators, not to mention the population observed: indeed, studies on adults with dyslexia remain marginal compared with those on children or adolescents.

### 3.3. Dyslexia and Writing Dynamics

People with dyslexia have some difficulties that persist in writing; several international studies confirm lexical, spelling and syntactic difficulties (among others, [13,14,15,16,17,18,19,20,21,22,23,24,25,26,27,28,29]). Concerning the dynamics of writing, there are few studies focusing on on-line indicators in people with dyslexia. There is no consensus concerning significant differences for handwriting speed between subjects with or without dyslexia among studies on dyslexic children [84] or dyslexic students [29]. It seems to also depend on the ages of the observed participants. The results of a study on children aged 9 reveal that handwriting speed (in terms of the physical distance covered by the pen divided by the time spent writing) is the same for both children with and without dyslexia [45,84], and that the slow writing of children with dyslexia is due to the production of pauses, which are longer and more frequent [84]. According to Sumner and Connelly [29], there is no significant difference between students with or without dyslexia, either in terms of handwriting speed (the same definition as [84]) or pauses. However, a recent study on French dyslexic teenagers [68] reveals that their handwriting speeds are below those found for typical middle-schoolers of the same age [85,86] and are more in line with the handwriting speeds of 11-year-olds. Few studies take into consideration pressure as an on-line indicator. But we can learn that the pressure that children with dyslexia (around eleven-and-a-half years old) exert on their pencil when they write letters of the alphabet or their first and last names is not different from that exerted by children of the same age without dyslexia [45]. The authors also conclude that, at this age, proprio-kinaesthesia skills are preserved, even for children with dyslexia. Likewise, Sumner et al. [84] provide one of the very few studies that include word rate in their indicators. They conclude that children with dyslexia (9-year-olds) wrote the same number of letters per minute in an alphabet task but fewer words per minute, compared to children without dyslexia. Similar results were found for students with dyslexia, concluding that they produce fewer letters per minute [51] or fewer symbols in 90 s [16] than control students. 

As for pauses and bursts, they reveal some differences between subjects with and without dyslexia. For instance, teenagers with dyslexia produce an atypical transcription of words in terms of on-line indicators during written production, with a slower handwriting speed, a smaller length of writing passages without pause, or with longer pauses between words and punctuation than typical teenagers [68,84,87]. The authors conclude that the allocation of cognitive resources is modified by cognitive hindrances, and that the process of word transcription can slow down or disorganize other higher-level cognitive operations. These conclusions confirm those of Galbraith and collaborators [88], who showed that students with dyslexia produce longer pauses within and between words than control students, and concluded that persistent difficulties with low-level processes interfere with high-level processes. Nevertheless, some studies on the writing processes of students with dyslexia do not support this view, specifically concerning pause times [29], suggesting that the transcription process (fluency of handwriting/writing) is not more hindered by spelling difficulties [29]. Moreover, previous studies reveal a difference between teenagers (12-year-olds) with and without dyslexia, also concerning bursts: the written production of dyslexic adolescents is regularly suspended by long pauses (threshold at 2s), which is less the case for adolescents without dyslexia [68]. The question is also to know, in the present paper, if university students with dyslexia are able to have long moments of production without a long pause, or if they rather have short moments interspersed with pauses of varying lengths [45].

## 4. Hypotheses

Most of the existing studies on writing focus on off-line indicators, like lexical choices, spelling or syntactic choices. In this present study, we examine the written language production of subjects with and without dyslexia matched for gender, age, and university level, by taking into consideration on-line indicators. 

First, some general on-line indicators are observed, such as handwriting speed, pressure and word rate. 

Second, pause duration is observed according to the location of pauses. We focus on pauses before words because this population is known to have persistent difficulties with spelling (see Part 2) and we take into account several linguistic features: length, consistency, frequency and grammatical class. If these features do not have an impact on the lexical choices of students with dyslexia [22], they can have an impact on the management of writing activities. 

Given the evidence concerning on-line indicators (see Part 3.2), we predict that the students with dyslexia have the same handwriting speed and pressure as the control students, which would be in line with previous work—H1 [29,45]; but, as found in earlier studies [16,51], as they do not have the same word rate, we expect that students with dyslexia produce fewer words per minute than students without dyslexia—H2; finally, moreover, based on previous work on pauses during a writing activity [29,45,68,84,87,88], they display different pause durations than the control group regardless of their location—H3: we expect that pause durations are longer for students with dyslexia before longer words, words with a lower degree of spelling consistency, rare words, or words belonging to grammatical classes involving inflection (nouns, verbs, and adjectives), such as agreement in gender and number, compared to students without dyslexia.

## 5. Method

### 5.1. Population

Data have been collected as part of a project concerned with the inclusion at university of French students with dyslexia. More precisely, data were collected in Lyon (France), from March 2015 to March 2016. This project implies several steps: (1) two online questionnaires [26]; (2) a speech and neuropsychological assessment [89]; and (3) a psycholinguistic task (production of four oral and written texts). For this article, only the written part of our psycholinguistic data are analysed and presented. As said before, the two populations of students are matched for gender, age and school level (Table 1).

The students with dyslexia were diagnosed during childhood; they have associated dysorthographia and received speech therapy. All students were monolingual native French speakers. Moreover, they all attended school in France. The participants all received an explanatory leaflet and signed an informed-consent form. These documents explained the project, the tasks they would have to carry out, and gave an update on the protection of their data. Exclusion criteria excluded people with any other disorders, or visual or hearing impairments.

### 5.2. Protocol

We propose to explain only the step of the psycholinguistic task, because the present paper focuses on the data from this step within the entire protocol (see [4,22,26] for further information on the global methodology). As said previously, the second step of the project was a speech and neuropsychological assessment implying written language processing tests in reading and writing (decoding, spelling and comprehension tests), meta-phonological, neuropsychological, visual–attentional (visual search test and visual and auditory orientation tests) and memory (short-term and auditory–verbal working memory) tests; for more information, you can consult previous work by Mazur et al. [4,22,89]. This enabled us to certify the dyslexia–dysorthographia of all the participants who declared that they had the disorder. The third step was the psycholinguistic task. The students were asked to produce four texts [90]: spoken and written narrative, and spoken and written expository. For the expository condition, we asked them to write a short essay about problems between people. For the narrative conditions, we asked them to report one of their personal experiences of conflict. Data were collected in two sessions (Table 2), one week apart. In the first session, they watched a three-minute wordless video depicting various conflict situations occurring at school (the Spencer project, R. Berman). They were then asked to produce a narrative or expository text, both in written and oral form. In the second session, the participants were asked to produce the other two texts. Between the productions of the written and oral texts, they completed a language questionnaire. The order of production of these texts was counter-balanced in order to control any sequential impact. The individuals were evenly divided into four passage orders, as illustrated in the following table. 

Thus, for instance, if a participant belongs to pass order A, during the first week, he watched the video then produced an oral narrative text. The experimenter then asked the subject to fill in a questionnaire and the participant then produced a written narrative text. A week later, he returned and produced an oral expository text, filled in another questionnaire, and produced a written expository text. 

There was no instruction about the duration of this task. They could take as much time as they wanted to write and proofread their texts. 

### 5.3. Collection and Exploitation of the Written Data

Subjects were asked to write by hand on a paper sheet laid on a digitizer tablet (Wacom Intuos 3, Wacom Company, Saitama, Japon), with no specific instructions regarding spelling, reviewing, correcting, etc. For written-text acquisition, we used the Eye and Pen© software (2.0 version) [91] and transcribed according to the CHILDES conventions, then exported into the CLAN software for the off-line analyses (see, for instance, [23,27,28,92]). For this study and the analyses of the on-line indicators, the data were coded in Eye and Pen© software. The productions were divided into clauses and terminal units (TUs, corresponding roughly to sentences) [92,93]. The corpus of written texts includes 86 written texts (43 expository and 43 narrative texts). For this study, narrative and expository texts have not been differentiated, and text type is not an independent variable. 

We reproduce below, from one of our preceding papers ([26], p. 9), the Table 3 which gives some information on the length indicators of the written texts of the corpus.

As said in that paper, “ANOVA analyses show that the differences in length between students with dyslexia and control students are not significant in the number of words (F(1.39) = 0.089, *p* = 0.767), of clauses (F(1.39) = 1.842, *p* = 0.183), of Units T (F(1.39) = 2.501, *p* = 0.122) and of clauses per Unit T (F(1.39) = 0.773, *p* = 0.385), [just like the] differences in the time duration (duration of written production, F = 2.07; *p* = 0.164 > 0.1)”.

### 5.4. Data Analysis

The main objective of the present paper is to link two types of indicators: on-line and off-line indicators. Firstly, three on-line indicators are analysed, which reflect the dynamics of the individual’s writing: handwriting speed, pressure and word rate, performing specific statistics (See Part 6.1). Handwriting speed is defined as the average speed of pen movements on the tablet (cm per second), in accordance with previous works [50,68,80], and is provided by the Eye and Pen© software. This indicator includes the cumulative length of all traces (in cm) divided by their cumulative duration (in seconds). Every move occurring when the pen is held up or down, and thus all pauses, are excluded. Pressure corresponds to the pressure that the writer exerts on the pen when he/she is writing [45]. Word rate corresponds to the number of words per minute, including pauses. 

Secondly, the duration (given in milliseconds (ms)) and location of pauses are analysed according to the linguistic features of the word that they precede. For each pause preceding a word, the following linguistic features are taken into consideration (inspired by a previous study and based on a processing chain specified in Mazur, Quignard and Witko [22]):

1. Word length: “short words: 1 to 4 letters; medium words: 5 to 7 letters; long words: more than 8 letters” ([22], p. 8; based on the Lexique and Manulex databases).
2. Graphemic and phonetic properties: the indicator of phoneme/grapheme consistency (based on [94]), defined as the degree of complexity of the spelling conversion. The phoneme/grapheme consistency of a word is the mean phoneme/grapheme consistency of all graphemes composing that word. Sublexical tables for French have been made available by the authors of [94] on their website (https://www.manulex.org, accessed on 10 January 2024; “Manulex-infra”). The degree of transparency and regularity of the spelling of a word, for instance, the word “monsieur” has, in French, a high level of consistency: it is an irregular word. The most consistent or transparent words have an indicator close to 1; the most difficult or opaque words have an indicator close to 0. The global scale of consistency has been divided into 4 subsets, according to the quartiles of the distribution of the entire Manulex database. Words are given a low level of consistency when their indicator is lower than 0.717. A high level of consistency relates to an indicator greater than 0.874. In between, two intermediate subsets are divided around the median of the distribution (0.807).
3. Frequency properties: frequency of lemmas. The lemma of a word is the form that is used as an entry in dictionaries. In inflected languages like French, these are singular masculine forms for nouns and adjectives, and infinitive forms for verbs (based on the Lexique.org, a French lexical database, based on French movie subtitles, number of occurrences per million). Words are divided into four subsets, according to the quartiles of distribution of the entire Lexique database. The three pivotal values are 0.12, 0.94 and 6.85.
4. One of the ten following grammatical classes: abbreviation, adjective, adverb, conjunction, determiner, noun, preposition, pronoun, verb and punctuation marks.

### 5.5. Statistical Analysis

In this section, several types of results are presented: firstly, specific statistics concerning on-line indicators, such as handwriting speed, pressure and word rate (6.1); secondly, analyses regarding the duration and location of pauses according to the linguistic features of the word that they precede (6.2). 

*Type of statistical analyses*. The main objective is to compare the means of the two groups of students (with and without dyslexia). We also decided, when the validity conditions were checked, to perform ANOVA tests, which are carried out between groups (students with dyslexia vs. control group) with the alpha level of 0.05. The effect size is called large when it is greater than 0.14, and moderate when it is greater than 0.06; otherwise, it is low. When the validity conditions of the ANOVA were not met, we performed a Kruskal–Wallis test.

*Outliers*. Because our experimental protocol allows individuals as much time as they deemed necessary to write their text, there may be considerable variability between individuals, and very long pauses. The literature highlights the effect of outliers on analyses (Field, 2013, [95]). Rather than removing them from the dataset, we made the methodological choice of neutralizing them by replacing them with the individual’s median value. We considered as an outlier a pause whose duration is longer than the median value plus 1.5 times the interquartile range in the pause distribution of each individual (not of the whole dataset), in order to respect inter-individual variability. We counted exactly 8.4% outliers in each group, which is quite fair and moderate.

*Test types*. As mentioned in the Method section, students were asked to write two text types: an expository and a narrative text. Tests are performed on the average of each measure for the two texts.

*Results tables*. The tables show the numbers, means and standard deviation of the two populations (students with dyslexia: DYS; students without dyslexia: CTRL), as well as the result of the statistical test. If the test is significant, the effect size is provided.

## 6. Results

### 6.1. Statistical Analyses of the On-Line Indicators

#### 6.1.1. Handwriting Speed

Table 4 presents the average handwriting speed for the students with and without dyslexia, and the mean comparison tests (ANOVA) performed, which reveal that the average handwriting speeds are very similar in both groups.

#### 6.1.2. Pressure

Table 5 presents the average pressure for the students with and without dyslexia, and the mean comparison tests (Kruskal–Wallis) performed, which reveal that the average pressures are very similar in both groups.

#### 6.1.3. Word Rate

Table 6 presents the average number of words produced per minute for the students with dyslexia and the control students, and the mean comparison tests (ANOVA) performed. 

Results reveal that the difference in average between the two groups is not significant.

### 6.2. Pause Duration According to the Linguistic Features of the Following Word

#### 6.2.1. Pause Duration Before Words According to Their Length

Table 7 presents the pause duration according to the length of the following word for the students with dyslexia and the control students, and the mean comparison tests (Kruskal–Wallis). 

**Figure 1:**
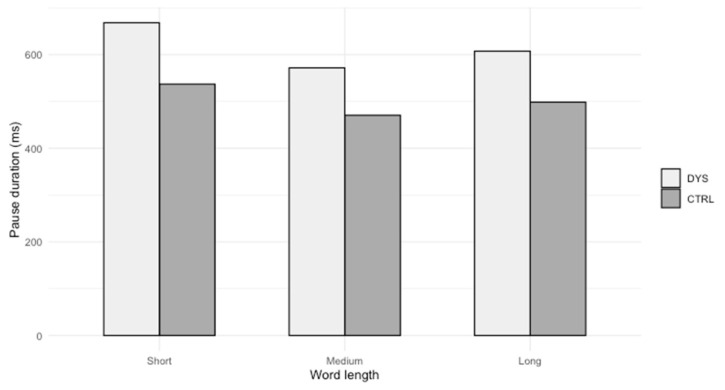
Pause duration before words according to their length.

Figure 1 illustrates these results.

Results reveal that students with dyslexia perform significantly longer pauses before short and long words, compared to students without dyslexia. 

#### 6.2.2. Pause Duration Before Words According to Their Spelling Consistency

Table 8 presents pause duration before words according to their spelling consistency for the students with dyslexia and the control students, and the mean comparison tests (ANOVA or Kruskal–Wallis). 

**Figure 2:**
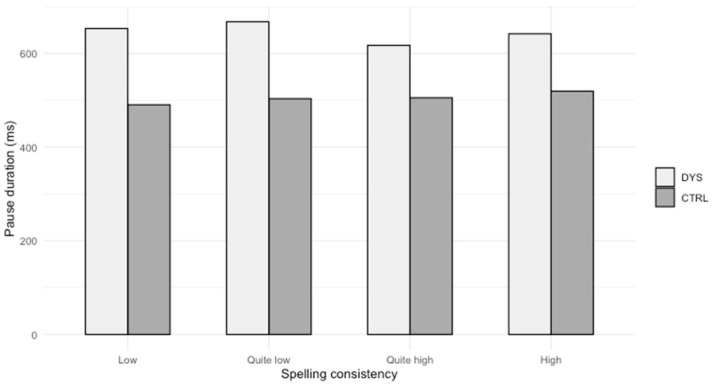
Pause duration before words according to spelling consistency.

Figure 2 illustrates these results.

Students with dyslexia make longer pauses before words with a low level of consistency (that means for words with an opaque spelling) than control students, and the effect size is large. Somewhat less significantly, they also make longer pauses than control students before words with a high level of consistency.

#### 6.2.3. Pause Duration Before Words According to Their Frequency

Table 9 presents pause duration before words according to their frequency, for the students with dyslexia and the control students, and the mean comparison tests (ANOVA or Kruskal–Wallis). 

**Figure 3:**
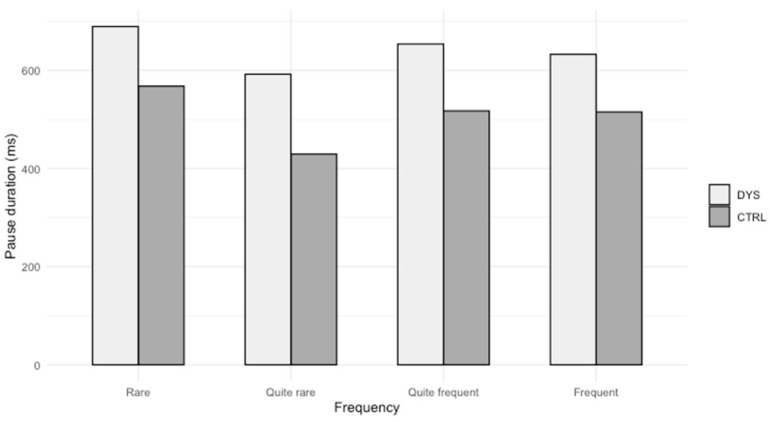
Pause duration before words according to their frequency.

Figure 3 illustrates these results.

The students with dyslexia make longer pauses than control students before frequent words, and the effect size is large.

#### 6.2.4. Pause Duration Before Words According to Their Grammatical Class

Mean comparison tests (ANOVA or Kruskal–Wallis) were performed on the pause duration before determiners (H_(1,43)_ = 6.51; *p* = 0.0107; moderate effect size: 0.134), nouns (H_(1,43)_ = 2.73; *p* = 0.09; not significant), verbs (H_(1,43)_ = 4.47; *p* = 0.034; moderate effect size: 0.084), adverbs (H_(1,43)_ = 4.68; *p* = 0.030; moderate effect size: 0.089), prepositions (H_(1,43)_ = 5.5; *p* = 0.018; moderate effect size: 0.11), adjectives (H_(1,43)_ = 1.20; *p* = 0.274; not significant), pronouns (F_(1,41)_ = 7.943; *p* > 0.007, significant; large effect size: 0.162), conjunctions (H_(1,43)_ = 1.30; *p* = 0.253; not significant) and punctuation signs (H_(1,43)_ = 8.50; *p* = 0.0035; large effect size: 0.183). Analyses reveal significant differences regarding the pause duration before determiners, verbs, adverbs, prepositions, pronouns and punctuation signs. 

Results reveal that pause duration is longer for students with dyslexia before the following:

1. Determiners (mean = 0.679; S.D. = 0.183) compared to control students (mean = 0.532; S.D. = 0.211);
2. Verbs (mean = 0.573; S.D. = 0.166) compared to control students (mean = 0.466; S.D. = 0.196);
3. Adverbs (mean = 0.650; S.D. = 0.223) compared to control students (mean = 0.517; S.D. = 0.180);
4. Prepositions (mean = 0.726; S.D. = 0.192) compared to control students (mean = 0.585; S.D. = 0.235);
5. Pronouns (mean = 0.690; S.D. = 0.212) compared to control students (mean = 0.523; S.D. = 0.176);
6. Punctuation signs (mean = 0.716; S.D. = 0.333) compared to control students (mean = 0.453; S.D. = 0.228).

**Figure 4:**
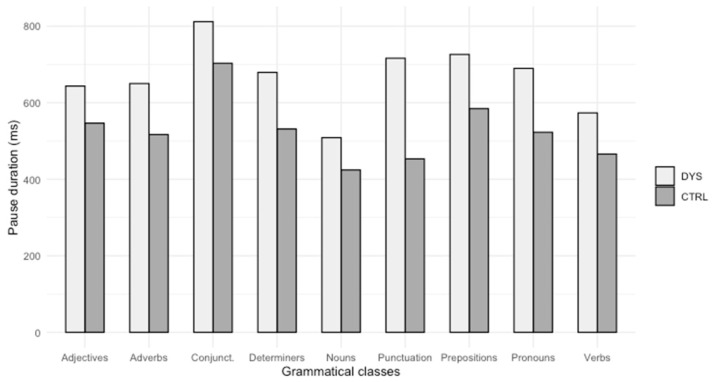
Pause duration before words according to their grammatical class.

Figure 4 illustrates these results.

## 7. Discussion

The main objective of this paper is to analyse some on-line indicators in the written language productions of students with and without dyslexia. As said in the Introduction, these indicators are based on the biomarkers of written production (here, pen movements). They may help to distinguish people with and without dyslexia, and maybe (with other bio-markers) to predict dyslexia. We decided to observe handwriting speed, word rate, and pause duration before words according to their length, frequency, consistency and syntactic class.

*Non-significant results*. Previous studies on English-speaking students with and without dyslexia conclude that students with dyslexia have the same writing dynamic as control students, as they produce the same distance of tracings per writing time [29]. Consistent with the literature, the present research found that the participants with dyslexia have the same handwriting speed and pressure as students without dyslexia (in line with hypothesis 1). This result supports previous observations concluding that spelling conversion-system difficulties do not hinder the transcription process (fluency of handwriting/writing) anymore [29], knowing that a slow handwriting speed may reflect planning or elaborating difficulties [41,81,82]. However, in contrast to earlier findings [16,51], our analyses reveal that students with dyslexia also have the same word rate as control students (thus refuting hypothesis 2): they produce approximately the same number of words per minute. But in those studies, word rate was calculated with the number of letters written per minute or the number of sentences written in two minutes. In our study and in another more recent study [29], when students were asked to produce a more complex written task (produce a written text) involving high-level processes, such as planning, translating and revising, students with and without dyslexia had an equivalent word rate. Finally, a few results reveal that pause duration before words is not significantly different for students with and without dyslexia, according to certain linguistic features, and more specifically before the following: words with a medium length, rare words, words with a rather high level of consistency, and before nouns, adjectives and conjunctions. We will discuss these results in the following part, when we present the associated significant results, which are much more numerous (hypothesis 3 is partially confirmed).

*Significant results*. Our results reveal that certain linguistic features of the following word have an impact on pause duration (hypothesis 3 is partially confirmed). Students with dyslexia make longer pauses than students without dyslexia before short and long words (by number of letters), but, as said previously, not before medium words. These results can be linked to a previous one concerning the number of spelling errors according to word length [22], which shows that students with dyslexia make many more errors on all categories of a word, regardless of its length. Moreover, those students are very sensitive to length and homophony (for instance, in French, “son” and “sont”): they make more errors on short homophonic words and on long non-homophonic words [25]. We can therefore cross-reference these previous results with the present one and conclude that students with dyslexia pause longer before these types of word, which also entail spelling problems for them. 

Moreover, as shown in Part 6.2.2, the dyslexic students make longer pauses before all words, regardless of their spelling consistency, except before words with a rather high level of consistency. In a previous study [22], the authors report that spelling consistency does not have any significant impact on the lexical choices and on the proportion of errors of students with dyslexia: they use the same types of word as control students, and they make the same number of spelling errors, regardless of their spelling consistency. Nevertheless, we can conclude with the new results of the present paper that this lexical choice has a cost: their pauses are longer for a same lexical panel compared to students without dyslexia. 

Concerning the impact of frequency on pause duration, as can be seen from Part 6.2.3, students with dyslexia make longer pauses before frequent words. This can be seen as an unexpected result. In fact, students with and without dyslexia take time to access lexical information (spelling) for rare words. Students without dyslexia do not have problems with frequent words; they have quick access to their lexical information, but it is a problem for students with dyslexia, who take more time. If “[they] use and write the same types words” as control students ([22], p. 16), here again, it has a cognitive cost which is reflected by longer pauses.

Finally, we wanted to see if the syntactic class of words can have an impact on the pause durations of students with dyslexia. Results reported in Part 6.2.4 reveal that, contrary to our expectations (H3-OH3), the students with dyslexia make longer pauses before words belonging to grammatical classes that do not involve lexical inflection: determiners, adverbs, and prepositions, and before pronouns and verbs. We expected longer pauses before nouns and adjectives, which involve lexical inflection, but this is not confirmed by statistical analyses. Nevertheless, we can see that words with grammatical inflection generate longer pauses for students with dyslexia (verbs and pronouns), just like grammatical words (determiners, adverbs and prepositions). These results can be discussed in the light of previous results [22,25], which show that students with dyslexia have more difficulties in handling short words and that these short words are often associated, in French, with a homophonic feature. So, students with dyslexia “have more difficulties with short and homophonic words or long and non-homophonic words” ([22], p. 16). This study supports evidence from these previous observations: longer pauses before these types of words can reflect difficulties in planning and translating their own written text. In a previous work [4], the authors conclude that students with dyslexia make longer pauses in general and between words. This finding is contrary to another study [29], where it was suggested that there is no significant difference between students with and without dyslexia, concerning pause duration. Longer pauses can reflect difficulties; indeed, it is commonly accepted that the longer pauses are before longer units. But here we are talking about pauses before small units: words. So, these long pauses before determiners, adverbs, prepositions, pronouns and verbs, may indicate some difficulties in processing these units, especially as students with dyslexia also make a lot of mistakes with these words, in particular the shorter ones [22,25]. This can be due to persistent difficulties with spelling, and also an unautomated spelling that still requires important cognitive resources. This impacts writing fluency: students with dyslexia need time to access the lexical information of words, and thus to handle transcription [68]. Finally, the present study reveals that pause duration before punctuation is longer for students with dyslexia than for students without dyslexia. A comparison of the findings with those of previous analyses [4,26] confirms that students with dyslexia need time to handle punctuation, which involves considerable syntactic planning [41]. The management of punctuation is deficient in students with dyslexia: the duration of their pauses is long before these marks, and they make a lot of punctuation errors [26]. This deficiency may reveal deeper difficulties, in the sense that commas and periods play an important role in textual cohesion, which corresponds to an early step of the activity of writing. We can conclude that longer pauses before punctuation marks can indicate a slowdown in their processing, and therefore a certain difficulty in managing them. As part of high-level processes, punctuation management is also strongly impacted by the lack of automation in spelling conversion.

## 8. Conclusions

The study set out to investigate the impact of dyslexia on written activities. To do so, we used biomarkers related to writing (movements directly linked to the pen) to establish indicators enabling us to observe and analyse the impact of dyslexia. We decided to work on two types of on-line indicators: indicators directly linked to the graphomotor dynamic of the activity, and pause duration according to the syntactic class of the word following the pause. The present study is one of the first attempts to thoroughly examine pause duration in a link with linguistic features of the word following the pause in question. The findings make several contributions to the current literature and to the care of students with dyslexia. 

First, this study confirms that the transcription level of the writing process, and more specifically spelling, remains costly, instead of being automated with age and experience [42,96]. This lack of automation entails that cognitive resources are shared between the three levels of writing: planning, translating and reviewing [38,60,82,96]. If cognitive resources continue to be allocated to the transcription level (spelling), the other levels, i.e., the more costly [38,60,82,96], are negatively impacted and lack resources. That can be reflected in an important proportion of spelling or punctuation errors [22,26], for instance, or in longer pauses before little units, reflecting a slowdown in the processing, as shown in the present study. Moreover, our study confirms that students with dyslexia master the graphomotor aspects of writing (the same handwriting speed, pressure and word rate as students without dyslexia), and that these motor aspects do not lead to a different dynamism or flow management in their written production. However, students with dyslexia still have important difficulties in writing, and their lack of automation in spelling has consequences on the transcription and planning process, even in adulthood.

Second, pause duration before words varies according to their linguistic features. Results show that according to length, spelling consistency, frequency and syntactic class, the length of pause durations can vary. Moreover, dyslexic students make longer pauses before grammatical words and words involving grammatical inflexion (verbs and pronouns). These types of word cause more problems for them than lexical words. This result is interesting and it should be taken into consideration in the remediation of people with dyslexia.

Thirdly, our study provides additional indications that will help to complete the dyslexia diagnosis and adapt the remediation as best as possible: bio-markers of written production can give indications about dyslexia, its degree of severity, its impact on writing (and not only on reading), and reveal an atypical management of written productions as well as the cognitive processes involved and impacted. The use of graphic tablets, and observation and analysis of on-line indicators, could be integrated into speech therapy practice; indeed, these on-line indicators are not detectable during a conventional work session with a patient [68,80] (Brun-Henin et al., 2012; Witko and Chenu, 2019), whereas they give interesting and important information about the management of writing. 

## 9. Limitations

One source of weakness in this study concerns the experimental protocol, and this could be the absence of a test to verify the automation of graphomotor gestures. Indeed, we could have planned a task focusing on the automation of graphomotor movements. Nevertheless, the international literature seems to attest to the fact that graphomotor skills are automated, even in individuals with dyslexia, when adult writers are concerned. If it is an aspect that needs to be taken into consideration for studies on children (among others [97]), this is not relevant for adults [43,44]. Another limitation of the present study concerns our statistical choices. Indeed, giving the subjects as much time as they want to write their text allows them to pause for as long as they feel is necessary. This leads to a high degree of variability in the duration of pauses (which does not affect writing speed). As a result, the distributions of pauses are affected by numerous outliers which prevent statistical analyses based on normal distributions (ANOVA) from being validly employed. To obtain results without distorting the distributions too much, we have chosen to consider as outliers pauses that are much longer than those used in the literature (an interquartile range of 3 instead of 1.5 IQR). It is likely that a non-spontaneous writing task (such as copying) would produce less problematic distributions.

## 10. Perspectives

One of the other biomarkers of dyslexia is eye-movement tracking, which can be observed through indicators such as ocular saccades. These indicators are already used in some reading experimentations, for children and adults, with and without dyslexia (see, for instance, the works of Bucci, M.-P.). However, these indicators are not widely used to observe handwriting, probably because the devices that have been used until now are rather invasive. Indeed, eye trackers involve immobilising or reducing head movements, in order to capture eye data. That said, a new project involves collecting eye data in addition to data relating to pen gestures (DysTracker project, scientific leaders: Mazur and Quignard, funding: LabEx ASLAN and Ecole Normale Supérieure de Lyon), using a new and less invasive device designed by the start-up Sierra. Now that the technological aspects and limitations have been overcome (by linking the software linked to the eye tracker and the graphic tablet that collects pen gestures), the data collection will soon begin. Taking into consideration two biomarkers of dyslexia sounds promising, and will provide new keys to a better understanding of dyslexia and to improve patient detection and care.

## Figures and Tables

**Table 1:** Description of the subjects who participated in the psycholinguistic task.

	Students with Dyslexia	Control Students
Mean age	21.7	21.8
Standard deviation	2.8	2.9
Age (Min–max)	18.1–28.5	18.1–28.9
Total number of participants	21	22
Gender	9 women/12 men	10 women/12 men
University level		
Bachelor	15	15
Master	4	5
PhD	1	1
Other	1	1

**Table 2:** Order of production (Q = Questionnaire).

	Session 1	Session 2
A	Narrative spoken–Q–Narrative written	Expository spoken–Q–Expository written
B	Narrative written–Q–Narrative spoken	Expository written–Q–Expository spoken
C	Expository spoken–Q–Expository written	Narrative spoken–Q–Narrative written
D	Expository written–Q–Expository spoken	Narrative written–Q–Narrative spoken

**Table 3:** Length indicators for the written texts according to the text type and group (standard deviations are given in brackets; Dys. = students with dyslexia, Con. = control students).

	Expository Texts		Narrative Texts	
	Dys.	Con.	Dys.	Con.
Mean duration of production (in minutes)	13.85 (8.60)	11.02 (8.16)	11.77 (7.60)	9.01 (4.94)
Number of words per text	198.2 (101)	181.1 (139)	207 (131)	181 (112)
Number of clauses per text	25.8 (12.7)	25 (17)	30.7 (20)	27 (16)
Number of TUs per text	12 (5)	12 (9.4)	14.7 (9.9)	13.8 (8.2)
Number of TU per clause	2.1 (0.5)	2.2 (0.4)	2.1 (0.3)	2.04 (0.5)

**Table 4:** Average handwriting speed (measured in centimetres per second).

Group	N	Mean	SD.	ANOVA
DYS	21	2.95	0.737	F_(1,41)_ = 0.01; *p* = 0.919
CTRL	22	2.97	0.553	

**Table 5:** Average pressure.

Group	N	Mean	SD.	Kruskal–Wallis
DYS	21	240	81.7	H_(1,43)_ = 0.577; *p* = 0.452
CTRL	22	225	42.2	

**Table 6:** Word rate (calculated in words per minute).

Group	N	Mean	SD.	ANOVA
DYS	21	17.6	4.05	F_(1,41)_ = 3.412; *p* = 0.072
CTL	22	20.1	4.83	

**Table 7:** Pause duration before words, according to their length.

Group	N	Mean	SD.	Kruskal–Wallis	Effect Size
Pause duration before short words					
DYS	21	0.668	0.183	H_(1,43)_ = 5.00; *p* = 0.0254	0.0975 (moderate)
CTL	22	0.537	0.199		
Pause duration before medium words					
DYS	21	0.607	0.179	H_(1,43)_ = 3.41; *p* = 0.0648	NS
CTL	22	0.498	0.172		
Pause duration before long words					
DYS	21	0.572	0.177	H_(1,43)_ = 4.27; *p* = 0.0389	0.796 (moderate)
CTL	22	0.470	0.177		

**Table 8:** Pause duration before words according to spelling consistency.

Group	N	Mean	SD.	ANOVA or Kruskal–Wallis	Effect Size
Pause duration before words with a low level of consistency					
DYS	21	0.653	0.169	F_(1,41)_ = 10.464; *p* = 0.002	0.203 (large)
CTL	22	0.491	0.161		
Pause duration before words with a rather low level of consistency					
DYS	21	0.668	0.203	F_(1,41)_ = 7.552; *p* = 0.009	0.156 (large)
CTL	22	0.504	0.190		
Pause duration before words with a rather high level of consistency					
DYS	21	0.617	0.272	H_(1,43)_ = 2.89; *p* = 0.089	
CTL	22	0.506	0.236		
Pause duration before words with a high level of consistency					
DYS	21	0.642	0.179	H_(1,43)_ = 5.22; *p* = 0.0224	0.103 (moderate)
CTL	22	0.520	0.209		

**Table 9:** Pause duration before words according to their frequency.

Group	N	Mean	SD.	ANOVA or Kruskal–Wallis	Effect Size
Pause duration before rare words					
DYS	21	0.689	0.305	H_(1,43)_ = 1.74; *p* = 0.187	
CTL	22	0.568	0.336		
Pause duration before rather rare words					
DYS	21	0.592	0.360	H_(1,43)_ = 0.91; *p* = 0.34	
CTL	22	0.429	0.168		
Pause duration before rather frequent words					
DYS	21	0.654	0.215	H_(1,43)_ = 5.11; *p* = 0.023	0.10 (moderate)
CTL	22	0.517	0.263		
Pause duration before frequent words					
DYS	21	0.633	0.176	H_(1,43)_ = 4.78; *p* = 0.0288	0.09 (moderate)
CTL	22	0.515	0.188		

## Data Availability

The data are not publicly available, due to the fact that it was specified to the participants that their written and spoken data would be not published.
